# Metabolomics Analysis Based on UHPLC-Q-TOF-MS/MS to Discriminate *Dictyophora rubrovolvata* from Different Geographical Origins of China

**DOI:** 10.3390/foods15081372

**Published:** 2026-04-15

**Authors:** Tingting Wang, Jinkun You, Juan Wang, Yayuan Deng, Qiuqiong Dai, Rong Hua, Dafeng Sun

**Affiliations:** 1 Yunnan Provincial Key Laboratory of Edible Fungi Germplasm Innovation and Functional Component, Kunming Edible Fungi Institute of All China Federation of Supply and Marketing Cooperatives, Kunming 650221, China; wtelain@163.com (T.W.); youjinkun0912@163.com (J.Y.); 18082756170@163.com (J.W.); yuejieqingfei@163.com (Y.D.); 15912456316@163.com (Q.D.); 2 Yunnan Academy of Edible Fungi Industry Development, Kunming 650221, China

**Keywords:** *Dictyophora rubrovolvata*, geographical origin, metabolomics, chemometrics

## Abstract

*Dictyophora rubrovolvata* is highly regarded and increasingly cultivated in China for its nutritional value, unique taste, and medicinal properties. However, the chemical composition of fresh *D. rubrovolvata* is unclear. This study applied a comprehensive metabolomic analysis of *D. rubrovolvata* to characterize and compare the metabolite profiles and identify significantly differential metabolites from three geographical origins in China. Ultra-high-performance liquid chromatography-quadrupole time-of-flight mass spectrometry (UHPLC-Q-TOF-MS/MS) combined with chemometrics was employed to conduct untargeted metabolomics analysis of fresh *D. rubrovolvata* samples collected from the Sichuan, Fujian, and Guizhou provinces in China. Among the 383 identified metabolites, lipids and organic acids were the predominant classes. There were notable variations in metabolite composition across the three geographical areas. The Sichuan (SC) group showed a high concentration of phospholipids, the Guizhou (GZ) group was characterized by specific oxidized lipids and bioactive benzenoids, and the Fujian (FJ) group showed elevated levels of the antioxidant ergothioneine. We identified 17 unique metabolites, including tryptophol, 12-oxophytodienoic acid, and various fatty acid derivatives, which may act as significantly differential metabolites for different origins. Analysis of KEGG enrichment indicated that the main metabolic pathways involved were tryptophan metabolism, glycerophospholipid metabolism, and phenylpropanoid biosynthesis.

## 1. Introduction

*Dictyophora rubrovolvata* is a highly valued edible mushroom belonging to the *Phallaceae* family, widely distributed in Asia [1]. In addition to its appealing taste and distinctive texture, *D. rubrovolvata* exhibits multiple physiological benefits, such as immune system modulation, antioxidant activity, and antitumor properties [2,3,4,5,6,7], which may be attributed to tis richness in polysaccharides, flavonoids, and other functional components [8,9]. In response to increasing commercial demands, *D. rubrovolvata* has swiftly shifted from being harvested in the wild to being cultivated through a modernized fungus-stick production system. This shift has led to the establishment of specialized production clusters in China, including regions such as Guizhou, Fujian, and Sichuan [10]. Considering the growing consumer concerns regarding its provenance and authenticity, establishing a reliable method for tracing the origin of *D. rubrovolvata* is essential. Recent investigations into polysaccharide structure have focused on those with specific glycosidic bonds, such as β-1,3-linkages, which are recognized for their potent immunomodulatory effects [1,7]. Many studies have been conducted to optimize the critical cultivation and production conditions of *D. rubrovolvata* [11]. An untargeted metabolomics analysis was performed to reveal the changes in metabolomic profiles of *D. rubrovolvata* under different drying treatments, with a particular focus on lipids, nucleotides, organic acids, and amino acids as key biomarkers [3]. Previous studies have shown that the browning of dried *D. rubrovolvata* stems can be traced to changes in metabolism during storage [12,13]. However, the metabolic mechanisms underlying the changes in the nutritional quality of major cultivars in nutritional quality are not yet completely clear.

Untargeted metabolomics utilizing ultra-high-performance liquid chromatography-mass spectrometry has been successful and widely applied in food science owing to its high throughput and broad coverage, which allows the determination of metabolic profiles and fingerprints [14]. UHPLC-QTOF-MS/MS is a well-established technique for untargeted metabolomics with high sensitivity and resolution. It has been used extensively for geographical authentication [15]. Geographical origin plays a role in the quality and chemical profiles of edible mushrooms, as environmental variations greatly impact the biosynthesis and accumulation of secondary metabolites [16]. Studies on high-value mushrooms have shown that abiotic factors, such as altitude, soil fertility, and microclimate, are the primary determinants of metabolomic profiles [17,18]. The application of multivariate statistical methods is essential for identifying significant patterns within high-dimensional metabolomic datasets of edible mushrooms, thus the utilization of *D. rubrovolvata* may reveal novel compositional markers linked to its geographical origin [19]. Chemometric tools improve classification accuracy by identifying essential metabolites that lead to differences in composition across regions and enhance the capability to differentiate mushrooms based on their geographical origin [20]. A previous study on *Butyriboletus roseoflavus* discovered that light intensity and humidity played crucial roles in modulating nucleotide metabolism and volatile flavor pathways [21]. The variability in aroma-active compounds of *Tricholoma matsutake*, such as 1-octen-3-ol and methyl cinnamate, is influenced by the maturity stage of the mushroom and its geographical location [22]. Currently, no research is available on the application of metabolomics technology to *D. rubrovolvata* grown in different regions of China.

This study utilized an untargeted metabolomics approach using UHPLC-Q-TOF-MS/MS to analyze and compare the metabolic profiles of *D. rubrovolvata* from three primary production areas (Sichuan, Fujian, and Guizhou) in China. To examine the discrimination among three geographical groups and identify significant differential metabolites, multivariate statistical analyses were conducted. This study provides a theoretical foundation for geographical chemical variations and quality characterization in the cultivation of *D. rubrovolvata*.

## 2. Materials and Methods

### 2.1. Materials and Chemicals

LC-MS grade methanol (MeOH), acetonitrile (ACN), and formic acid were obtained from Thermo Fisher Scientific (Rockford, IL, USA). Ultrapure water used throughout the experiment was obtained using a Milli-Q system (Millipore, Bedford, MA, USA).

We collected fresh *D. rubrovolvata* samples in July 2025 from the provinces of Sichuan (SC), Fujian (FJ), and Guizhou (GZ) of China. In each province, one representative commercial cultivation base from the major production area was selected for sampling: Chengdu, Sichuan Province (104.71° E, 30.42° N) (SC); Zhangzhou, Fujian Province (117.52° E, 24.98° N) (FJ); Bijie, Guizhou Province (105.87° E, 26.60° N) (GZ). All collections were conducted within the same harvest period to reduce seasonal effects on metabolite variation. At each cultivation base, a stratified random sampling approach was used to improve within-site representativeness. The cultivation area was divided into several sampling plots, and fruiting bodies were randomly collected from multiple positions across the site. Fifteen individual fruiting bodies were collected from each location. The fruiting bodies, which were disease-free and exhibited uniformity in color, size, and maturity, were sealed and stored at −20 °C until subsequent analysis.

### 2.2. Metabolite Extraction

A previous method [23] was used for this experiment with some modifications. Samples of fruiting bodies (80 mg) collected from three geographical areas were immediately frozen in liquid nitrogen and crushed into a fine powder. After adding 1000 μL MeOH/ACN/H_2_O (2:2:1, *v*/*v*/*v*) to the homogenized solution and centrifuging for 20 min (14,000× *g*, 4 °C), the supernatant was dried using a vacuum centrifuge (Eppendorf, Enfield, CT, USA). Samples were re-dissolved in 100 μL of ACN/H_2_O (1:1, *v*/*v*), centrifuged at 14,000× *g* and 4 °C for 15 min, and the supernatant was injected for LC-MS analysis.

### 2.3. UHPLC-Q-TOF-MS/MS Conditions

Metabolite separation was performed using an Agilent 1290 Infinity LC ultra-high-performance liquid chromatograph (Agilent Technologies, Santa Clara, CA, USA) with an AB Sciex TripleTOF 6600 quadrupole time-of-flight mass spectrometer (AB Sciex, Milford, MA, USA). A Waters ACQUITY UPLC BEH C18 column (1.7 μm, 2.1 mm × 100 mm) was used for separation and maintained at a temperature of 40 °C. The flow rate was set to 0.4 mL/min with an injection volume of 2 μL. Mobile phase A was composed of 25 mM ammonium acetate and 0.5% formic acid dissolved in water, while mobile phase B consisted of methanol. Gradient elution procedure was started with 5% B from 0 to 0.5 min, followed by a linear increase to 100% B from 0.5 to 10 min, and maintained from 10 to 12 min; from 12.0 to 12.1 min, a shift from 100% back to 5% B; from 12.1 to 16 min, 5% B. Throughout the analysis, samples were kept in an automatic sampler at 4 °C, and a random sequence was employed for sample analysis to mitigate the impact of instrument fluctuations. quality control (QC) samples were randomly positioned in the sample queue to assess data stability and system reliability.

The ESI source conditions were configured with Ion Source Gas1 and Gas2 at 60, curtain gas at 30, a source temperature of 600 °C, and an IonSpray Voltage Floating at ±5500 V. During MS-only acquisition, the instrument scanned the *m/z* range of 60–1000 Da with a TOF MS scan accumulation time of 0.20 s per spectrum. In auto MS/MS acquisition, the instrument was configured to capture data within the *m/z* range of 25–1000 Da with a product ion scan accumulation time of 0.05 s per spectrum. Utilizing information-dependent acquisition (IDA), the product ion scan was recorded with the high-sensitivity mode activated, and the parameters were configured with a collision energy (CE) of 35 V ± 15 eV, a declustering potential (DP) of 60 V for positive ions and −60 V for negative ions, exclusion of isotopes within 4 Da, and monitoring of 10 candidate ions per cycle.

### 2.4. Data Analysis

The raw MS data (wiff.scan files) were converted to MzXML files using ProteoWizard MSConvert prior to import into the open-source XCMS software v 3.4.2. Peak picking was conducted using the following parameters: centWave *m/z* of 10 ppm, peakwidth of c (10, 60), and a prefilter set to c (10, 100). The parameters for peak grouping were set as follows: bw at 5, mzwid at 0.025, and minfrac at 0.5. Isotopes and adducts were annotated using CAMERA (Collection of Algorithms of MEtabolite profile Annotation). Ion features were retained only if they had more than 50% nonzero measurements in at least one group. Metabolite identification was conducted by matching the *m/z* values (<10 ppm) and compound annotation was conducted by aligning retention time, accurate mass, MS/MS fragmentation spectra, and collision energy data with an in-house database supplied by Shanghai Applied Protein Technology. This database was constructed using reference information obtained from authentic standards. Annotation confidence was assessed in accordance with the Metabolomics Standards Initiative (MSI) guidelines. After normalization to total peak intensity, the processed data was subjected to multivariate data analysis. The consistency of QC samples was evaluated using Pearson correlation analysis and relative standard deviation (RSD).

The processed data were examined using the *R* package (version 3.3.3) and underwent multivariate data analysis, which included Pareto-scaled principal component analysis (PCA) and orthogonal partial least-squares discriminant analysis (OPLS-DA). To assess the model’s robustness, 7-fold cross-validation and response permutation testing were employed. The contribution of each variable to the classification in the OPLS-DA model was determined by calculating the variable importance in the projection (VIP) value. Student’s *t*-test was conducted to evaluate the significance of differences between the two groups. *p* value < 0.05 was employed as a criterion to identify significant alterations in metabolites.

## 3. Results and Discussion

### 3.1. Overview of Metabolites in D. rubrovolvata from Different Origins

The geographical origin of edible mushrooms depends heavily on their sensory quality and nutritional value owing to variations in climatic conditions and soil composition [24,25]. Chemometric analyses, with a focus on multivariate models, are frequently utilized in metabolomic research to examine the differences among vast quantities of omics data [26,27]. We employed an untargeted metabolomics approach utilizing UHPLC-QTOF-MS/MS to analyze and contrast the metabolite profiles of *D. rubrovolvata* from the provinces of Sichuan (SC), Fujian (FJ), and Guizhou (GZ). This study aimed to investigate the metabolic profiles specific to their origins and the chemical basis for quality variation.

**Figure 1:**
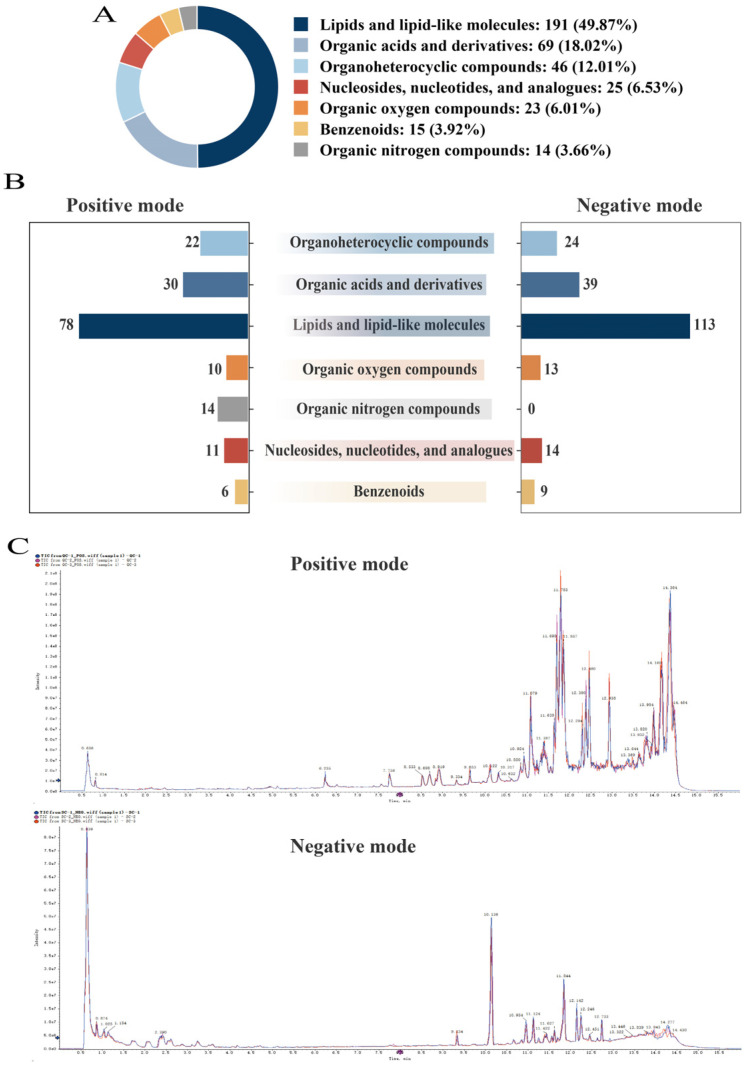
Overview of the metabolites in *D. rubrovolvata*. Classification pie chart of metabolites in samples from different geographical areas of China (**A**). Bar chart of metabolite classification in the positive and negative ion modes (**B**). The *x*-axis represents the number of metabolites in each class, and the *y*-axis represents the metabolite classification entries. Total ion chromatograms of QC in positive (POS) and negative (NEG) ion modes (**C**).

A total of 383 metabolites were identified across the three samples, with 171 metabolites detected in the positive ion mode and 212 in the negative ion mode (Figure 1A,B). Organic nitrogen compounds were examined exclusively in the negative ion mode. The total ion chromatogram of QC for *D. rubrovolvata* samples collected from three different regions in China demonstrated the reliability and uniformity throughout the data acquisition process, with no significant abnormal drift or batch instability observed (Figure 1C). A comprehensive evaluation of the QC results is presented in Supplementary Figure S1. The metabolomic profiles of SC, FJ, and GZ demonstrated uniform effectiveness and were comparable for further differential analyses. As shown in Figure 1, metabolites were classified into various categories. As the dominant category (49.87%, 191 metabolites) of *D. rubrovolvata*, lipids and lipid-like molecules in *D. rubrovolvata* are essential precursors for flavor development during processing [28]. In the composition of *D. rubrovolvata* categories, organic acids and their derivatives constituted 18.02% and organoheterocyclic compounds represented 12.01%. In this study, there were also biologically active and flavor-contributing compounds such as nucleosides, nucleotides, and analogues (6.53%), organic oxygen compounds (6.01%), and organic nitrogen compounds (3.66%). Organic acids and nucleotides are crucial because they are linked to the characteristic umami taste and nutritional roles of mushrooms [29].

### 3.2. Comparison of Metabolite Composition Among Three D. rubrovolvata Samples

**Figure 2:**
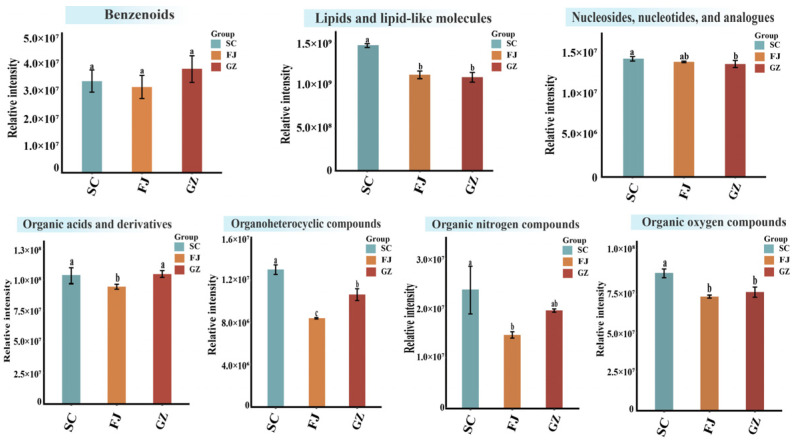
Relative intensities of different metabolite categories from different geographical areas (SC, FJ, and GZ). Data are presented as mean ± standard deviation (*n* = 3). Different letters in the bar chart indicate significant differences between samples (*p* < 0.05).

**Figure 3:**
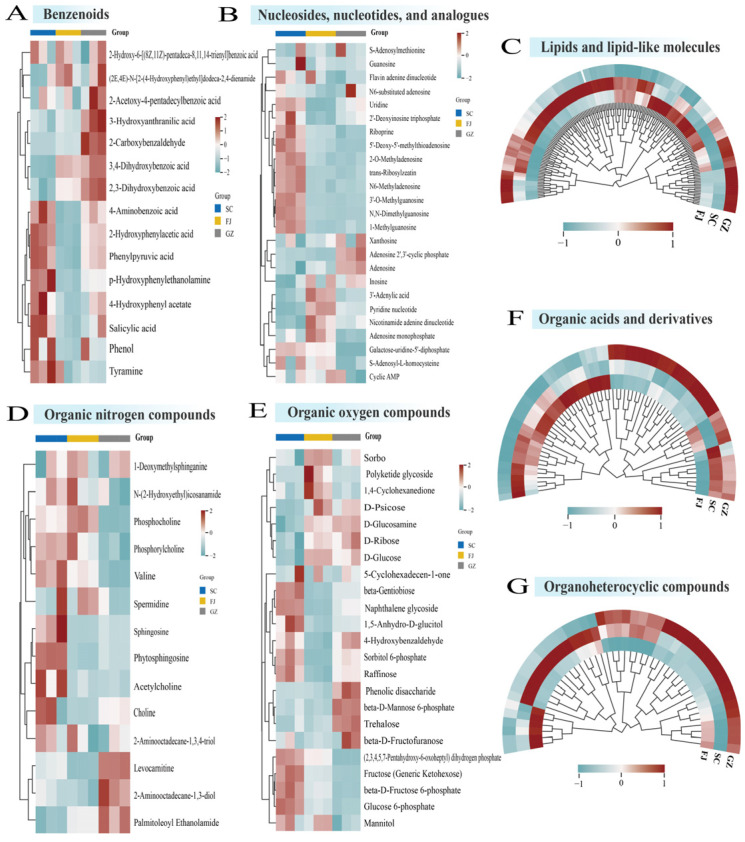
Heat map of different metabolite levels in *D. rubrovolvata* samples from three geographical areas. The relative content was calculated by comparing the relative intensities of the samples and is represented as a heat map after row normalization. Values above the mean are marked in red, and those below the mean are marked in blue. The depth of the color indicates the magnitude of the deviation from the mean.

To elucidate the differences in metabolites across the three geographical origins, we measured the relative intensities of the metabolites and presented the results using histograms and heatmaps (Figure 2 and Figure 3). Statistical analyses showed that samples from the three geographical origins differed significantly in terms of categories such as lipids and lipid-like molecules, organic acids and derivatives, organoheterocyclic compounds, and organic nitrogen compounds. Nevertheless, the total amount of benzenoids varied slightly in terms of the total relative content (Figure 2). Fifteen benzenoid derivatives were detected in three samples from different areas. The heatmap from the hierarchical clustering of benzenoid derivatives revealed unique metabolic patterns corresponding to each geographical origin (Figure 3A). The levels of phenylpyruvic acid, salicylic acid, and tyramine in SC were significantly higher compared to the other two groups. Salicylic acid serves as a signaling molecule during stress and is vital for initiating plant defense mechanisms [30]. In GZ, the relative content of 2-carboxybenzaldehyde was five times higher than that in SC, and the most prevalent compounds in GZ were 3,4-dihydroxybenzoic acid and 3-hydroxyanthranilic acid. Protocatechuic acid is an aromatic benzenoid derivative frequently identified in secondary metabolism [31]. The specific enrichment of these oxygenated derivatives suggests that the Guizhou cultivation environment may influence the accumulation of particular aromatic metabolitesin *D. rubrovolvata*.

Lipids and lipid-like molecules were the primary metabolites in all three samples, with 191 lipid molecules significantly affected by geographic origin. As shown in Figure 2, SC had a much higher total intensity of lipid molecules than FJ and GZ. *D. rubrovolvata* tends to spoil quickly because of its high moisture content, which facilitates the growth of harmful microorganisms and enzymes. Hot-air drying is commonly employed to extend the shelf life of food products. During thermal drying, lipids in mushrooms can generate distinct aromas and flavors through thermal or enzymatic reactions [32]. As illustrated in Figure 3C, SC was rich in phospholipids and essential fatty acids (e.g., phosphatidylcholine lyso 18:2 and various glycerophospholipids). Linoleic acid and alpha-linolenic acid in SC are known as unsaturated fatty acids to enhance nutritional value and aroma. Despite its lower total lipid content, GZ accumulated oxygenated lipids, such as 9-Hydroxy-10,12-octadecadienoic acid, and hydroxy-fatty acids. The increased levels of oxidized derivatives suggest that the metabolic processes in the Guizhou sample may favor the lipoxygenase pathway, which is linked to mushroom aroma profiles, such as C8 compounds [33]. Most lipid molecules found in FJ generally displayed the lowest levels, with relatively less active lipid metabolism.

The unique chemical profiles (nucleosides, nucleotides, and their analogues) in *D. rubrovolvata* are associated with umami-related flavor characteristics of edible mushrooms. We identified 25 metabolites in this class. The total abundance indicated a significant geographical gradient (Figure 2), with the highest accumulation in SC, followed by FJ and GZ. As 5′-nucleotides could act with amino acids to enhance umami perception [34], their abundance in SC suggests a stronger intrinsic umami potential. Heatmap visualization (Figure 3B) also revealed the metabolic biases contributing to these differences. N6-methyladenosine and UDP-Gal were highly enriched in SC than in FJ and GZ. The findings suggest that metabolic processes may reflect differences in nucleotide sugar metabolism and methylation-related cycles among samples from different regions. GZ contained a higher relative abundance of free adenosine than SC or FJ, highlighting a distinct nucleoside-related metabolic feature of this geographical origin [35].

Organic acids and amino acids are responsible for sour, sweet, and umami tastes. This class consisted of 69 molecules, and the outcomes showed significant variation based on geographical origin. SC and GZ exhibited consistently high levels overall, whereas FJ exhibited notably lower levels. Although SC and GZ had similar overall abundances, the heatmap (Figure 3F) highlighted two unique metabolic approaches associated with their taste quality. One of the most common metabolic strategies in GZ was succinic acid, known for its distinctive and intense umami flavor [36]. The citric acid content was nearly doubled in GZ compared to that in SC. *D. rubrovolvata* from Guizhou may possess a complex flavor profile characterized by strong acidity and savory notes owing to high levels of succinic and citric acids. SC had a higher glutamine content than FJ and GZ. Glutamine is typically characterized by subtle sweetness, suggesting that Sichuan origin may contribute to a more balanced flavor profile [37]. FJ had the lowest amount of total organic acids, yet its ergothioneine content was double that of SC and GZ. Ergothioneine is an antioxidant derived from fungi that has protective effects on cells [38], suggesting that FJ may have a stronger antioxidant potential.

The organoheterocyclic compounds were ranked in the order SC > GZ > FJ, with all pairwise comparisons being significant (Figure 2). In total, 46 metabolites were detected in the three samples. And as shown in Figure 3G, the tryptophan content was lower in SC than in FJ and GZ, the downstream conversion products were higher in SC. The tryptamine and tryptophol levels were notably higher in SC than in GZ and FJ. Nicotinamide was found in the highest concentration in GZ, significantly exceeding the amounts present in FJ and SC. The same situation may be relevant for ascorbic acid.

Organic nitrogen compounds (e.g., alkaloids, amines, and sphingolipids) showed significant variation among the three *D. rubrovolvata* samples (Figure 2 and Figure 3D). SC contained the highest amount of nitrogenous compounds compared to FJ, with acetylcholine present in SC at ten times the amount found in both FJ and GZ. SC contained a greater amount of phytosphingosine, a representative sphingolipid-related metabolite reported in mushroom [39], than the other two areas. Levocarnitine was most distinctive, with GZ levels higher than SC and FJ. GZ contained a high content of palmitoleoyl ethanolamide, a naturally occurring fatty acid amide [40].

Figure 2 and Figure 3E present analyses of organic oxygen compounds, with particular emphasis on carbohydrates and their derivatives. SC had the highest carbohydrate biomass compared to GZ and FJ. In SC, the levels of glucose 6-phosphate and fructose 6-phosphate, acting as connecting metabolites between pentose phosphate and glycolysis pathways, were considerably increased. SC contained more gentiobiose, a disaccharide with a bitter taste. The presence of gentiobiose alongside sweet glutamine likely contributes to the complex flavor profile of Sichuan *D. rubrovolvata*. The relative content of trehalose was threefold higher in GZ than in SC. FJ had a lower overall content but was rich in D-glucose, which might directly lead to an immediate sweet taste and serve as a precursor for the Maillard reaction during heat processing.

### 3.3. Multivariate Statistical Analysis of the Metabolites in Different Geographical Regions

**Figure 4:**
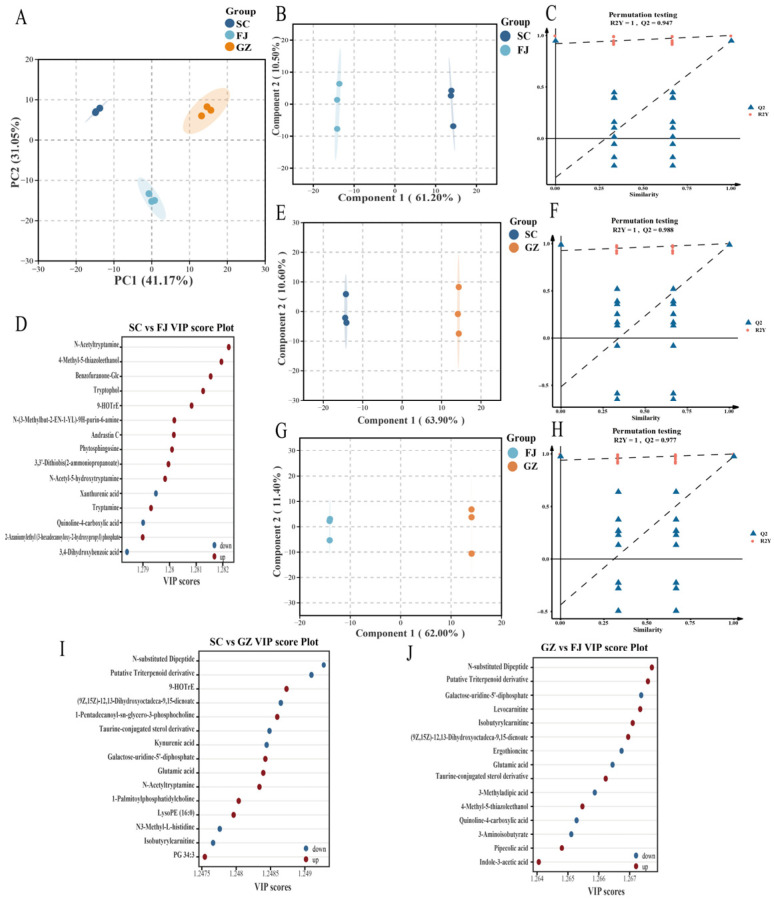
Multivariate statistical analysis of the metabolite profiles of *D. rubrovolvata* samples from different geographical areas. Score plot of PCA model (**A**). Score plots of OPLS-DA model for SC vs. FJ, SC vs. GZ, and GZ vs. FJ (**B**,**E**,**G**). 200 times permutation test plots for SC vs. FJ, SC vs. GZ, and GZ vs. FJ (**C**,**F**,**H**). Top 10 compounds with the highest VIP scores for SC vs. FJ, SC vs. GZ, and GZ vs. FJ (**D**,**I**,**J**).

Distinguishing samples based on solely raw data is challenging because of the complexity and high dimensionality inherent in metabolomics data [41]. Multivariate statistical analyses were conducted to detect differences between datasets and to pinpoint distinct metabolites from various angles. Principal component analysis (PCA), an unsupervised technique for examining multivariate datasets, was initially utilized to observe grouping patterns and identify outliers within the dataset [42]. Figure 4A effectively differentiates between samples from various geographical origins using a PCA score plot. The first principal component (PC1) accounted for 41.17% of the variance, and the second principal component (PC2) explained 31.05%. Samples from Sichuan (SC), Fujian (FJ), and Guizhou (GZ) exhibited tight clustering within their respective groups, demonstrating high reproducibility in both the extraction and detection processes. These three groups were distinctly separated in orthogonal space due to significant geographical influences on the metabolic profiles of *D. rubrovolvata*.

We utilized OPLS-DA to enhance the identification of differential metabolites through pairwise comparisons. This approach is particularly useful for eliminating variations that do not directly contribute to class separation [43]. Figure 4B,E,G reveal clear distinctions among groups in all pairwise comparisons (SC vs. FJ, SC vs. GZ, GZ vs. FJ), and the statistical parameters of the models are summarized in Table S1. The model demonstrated exceptional fitness parameters with high predictability and reliability between the model of SC vs. FJ (R^2^Y_cum_ = 1.00, Q^2^_cum_ = 0.947) and SC vs. GZ (R^2^Y_cum_ = 1.00, Q^2^_cum_ = 0.988). These metrics further highlighted its reliability (R^2^Y_cum_ = 1.00, Q^2^_cum_ = 0.977) in the GZ vs. FJ group. To evaluate the validity of the models and eliminate the possibility of overfitting, permutation tests were conducted 200 times (Figure 4C,F,H). Pairwise cross-validation conducted among the three model groups demonstrated a high level of reliability and appropriate fit within an acceptable range.

The key factors leading to this separation were identified using VIP scores. In the pairwise comparisons of SC vs. FJ, SC vs. GZ, and GZ vs. FJ, the top 10 differential metabolites with VIP > 1 were identified (Figure 4D,I,J). N-acetyltryptamine, 4-Methyl-5-thiazoleethanol, tryptophol, and tryptamine were significantly upregulated in SC vs. FJ (Figure 4D). Certain phenolic compounds, such as 3,4-dihydroxybenzoic acid and xanthurenic acid, were downregulated. In the comparison of SC vs. GZ (Figure 4I), SC had significantly higher contents of n-acetyltryptamine, glutamic acid, PG 34:3, 9-HOTrE, and galactose-uridine-5′-diphosphate, while GZ demonstrated higher contents of kynurenic acid and N3-methyl-L-histidine. Comparing GZ to FJ (Figure 4I), GZ showed an increase in levocarnitine, pipecolic acid, and indole-3-acetic acid, whereas FJ was characterized by a notable presence of nutraceutical and flavor compounds, particularly ergothioneine and glutamic acid.

We analyzed the differences in metabolite content by examining fold change (FC) values in pairwise comparisons using a threshold of FC >2.0 or <0.5. The results were visualized using rank-ordered dynamic distribution plots (Figure S2A–C) for the comparison groups SC vs. FJ, SC vs. GZ, and GZ vs. FJ, highlighting the top 10 metabolites that exhibited significant regulatory changes. Tanshinone IIA, L-malic acid, tryptophol, and tryptamine increased in SC (Figure S2A), whereas FJ showed enriched phospholipids with decreased PE 32:1 and PE 34:2. In the comparison between SC and GZ (Figure S2B), acetylcholine and D-pantothenic acid were found to be significantly elevated in SC. In contrast, GZ showed higher relative levels of FA 18:3+2O and PE 32:1. GZ showed an increased presence of L-malic acid and terpenoids (e.g., a derivative of picras-4-en-16-one) in GZ vs. FJ (Figure S2C), and FJ had a greater concentration of indole-3-acetaldehyde and PI 34:1.

**Figure 5:**
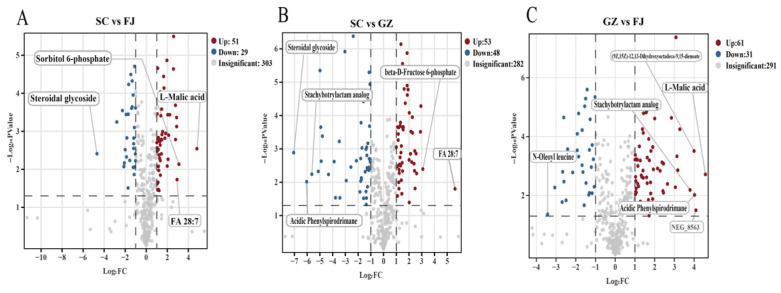
Variation in metabolites in pairwise comparison of *D. rubrovolvata* samples from different geographical areas. Volcano plots of metabolites in SC vs. FJ (**A**), SC vs. GZ(**B**), and GZ vs. FJ (**C**). Red dots indicate upregulated metabolites, and green dots indicate downregulated metabolites.

To statistically identify the most significant differential metabolites, volcano plots were generated by combining the fold change (Log_2_FC) with the significance level (−Log_10_ *p* values) using the *p* value derived from a *t*-test. The screening criteria were FC >2 or <0.5 and *p* < 0.05, with red points indicating significantly upregulated metabolites and blue points indicating significantly downregulated metabolites in the volcano plots (Figure 5A–C). There were 80 differential metabolites in SC vs. FJ (Figure 5A), with 51 exhibiting an increase (e.g., L-malic acid, sorbitol 6-phosphate, and FA 28:7) and 29 exhibiting a decrease (e.g., steroidal glycoside). A total of 101 differential metabolites were identified in the SC vs. GZ comparison (Figure 5B), of which 53 were upregulated (e.g., FA 28:7 and β-D-Fructose 6-phosphate) and 48 were downregulated (e.g., acidic phenylspirodrimane and stachybotrylactam analog). In the comparison between GZ and FJ (Figure 5C), 92 potential differential metabolites were identified. Among these, 61 were significantly upregulated (e.g., L-malic acid, stachybotrylactam analog, and acidic phenylspirodrimane), whereas 31 were downregulated (e.g., n-oleoyl leucine).

**Figure 6:**
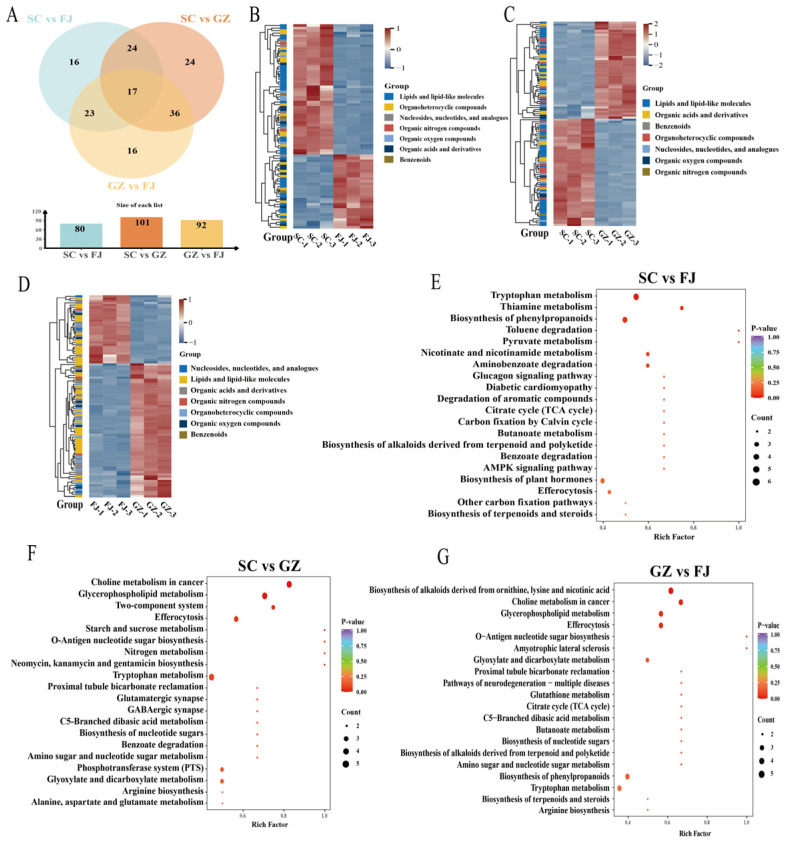
Screening and comparison of differential metabolites. Venn diagram of differential metabolites in SC vs. FJ, SC vs. GZ, and GZ vs. FJ (**A**). Heat map analysis and categories of differential metabolites in SC vs. FJ (**B**), SC vs. GZ (**C**), and GZ vs. FJ (**D**). KEGG enrichment pathway analysis of differential metabolites in SC vs. FJ (**E**), SC vs. GZ (**F**), and GZ vs. FJ (**G**). In the plot, each bubble (representing a metabolic pathway) and the abscissa indicate the size of the factors affecting the pathway (larger bubbles represent larger impacts). The bubble color indicates the *p*-value of the enrichment analysis, and a lighter color indicates lower enrichment.

To identify key differential metabolites capable of distinguishing *D. rubrovolvata* samples from various geographical origins, stringent screening criteria were applied, including VIP > 1, FC > 2 or <0.5, and *p* < 0.05. As depicted in Figure 6A, the extent of metabolic divergence varied significantly based on geographical origin. The Venn diagram delineated distinct subsets of markers responsible for the overlapping metabolites observed across the comparisons. The comparison between SC and GZ demonstrated the highest number of 101 differential metabolites, followed by the GZ vs. FJ comparison with 92 differential metabolites and the SC vs. FJ comparison with 80 differential metabolites.

Hierarchical clustering analysis (HCA) was conducted on 80 differential metabolites identified in the SC vs. FJ comparison (Figure 6B and Table S2), comprising 34 lipids and lipid-like molecules, 18 organic acids and derivatives, 3 benzenoids, 6 nucleosides and analogues, and 19 organoheterocyclic compounds. SC exhibited higher contents of L-malic acid, FA 28:7, tryptophol, and tryptamine than FJ. 101 differential metabolites were identified in SC vs. GZ (Figure 6C and Table S3), including 47 lipids and lipid-like molecules, 3 benzenoids, 8 nucleosides and analogues, 19 organic acids and derivatives, 16 organoheterocyclic compounds, 2 organic nitrogen compounds, and 6 organic oxygen compounds. Ala-Leu, Gly-Leu, kynurenic acid, oxophytodienoic acid, 9-HPODE, and trehalose in GZ were significantly higher compared to SC. This study investigated the metabolic differences between GZ and FJ and identified 92 differential metabolites. Among these, there were 40 lipids and lipid-like molecules, 23 organic acids and derivatives, 7 organic oxygen compounds, 4 nucleosides and analogues, 3 organic nitrogen compounds, 13 organoheterocyclic compounds, and 2 benzenoids (Figure 6D and Table S4). FJ identified the accumulation of specific metabolites such as in-dole-3-acetaldehyde, glycero-3-phosphocholine, oxophytodienoic acid, and β-D-fructose 6-phosphate.

**Figure 7:**
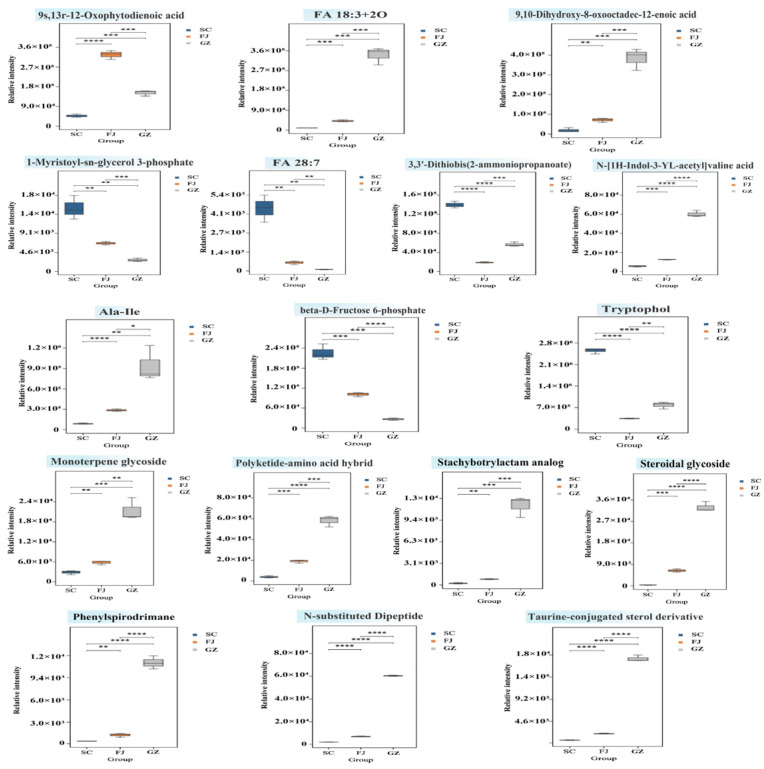
Boxplots of key differential metabolites between the three geographical origins of the *D. rubrovolvata* samples (Figure 7). * *p*  <  0.05; ** *p* <  0.01; *** *p* < 0.001; and **** *p* < 0.0001.

17 differential metabolites with significant variations across all three pairwise comparisons were identified through Venn diagram analysis (Figure 6A and Table S5). The conserved markers served as fundamental metabolic signatures that distinguished *D. rubrovolvata* from various geographical origins. Boxplots were created to illustrate the specific variation patterns of these key metabolites (Figure 7). Among the 17 differential metabolites, lipids and lipid-like molecules were the most prevalent, followed by derivatives of organic acids and organoheterocyclic compounds. FJ exhibited the highest abundance of 9s,13r-12-oxophytodienoic acid, followed by GZ and SC, with similar patterns observed for the long-chain fatty acids. FA 18:3+2O, 9,10-dihydroxy-8-oxooctadec-12-enoic acid, N-[1H-indol-3-yl-acetyl] valine, and Ala-Ile exhibited significant enrichments in GZ, with the oxidized fatty acid content in GZ being notably higher. Oxylipins are associated with membrane lipid peroxidation and signaling [44]. FA 28:7, 1-myristoyl-sn-glycerol 3-phosphate, 3,3′-dithiobis(2-ammoniopropanoate), β-D-fructose 6-phosphate, and tryptophol were identified as distinctive markers for SC, with their concentrations being significantly higher than those observed in FJ and GZ. Lysophosphatidic acids (e.g., 1-myristoyl-sn-glycerol 3-phosphate) are crucial intermediates in glycerophospholipid biosynthesis and contribute to cell proliferation [45]. The presence of glycolytic intermediates in SC indicates a highly active primary metabolism that generates sufficient energy to support rapid growth and biomass accumulation [46]. The lipid derivatives featuring furan and spiro-naphthalene structures also exhibited notable variations. To optimize the benefits of research, it is recommended that future studies incorporate a more comprehensive sample set and utilize independent sample sets in conjunction with targeted validation strategies. This methodology will more effectively assess the efficacy of these significantly differential metabolites in distinguishing geographic origins.

Metabolic and regulatory pathways provide organized and thorough insights into how biological functions are shaped by geographical origin [47]. To further elucidate the biological functions of the differential metabolites, we investigated pathway enrichment analysis between SC and FJ (Figure 6E). Tryptophan metabolism, phenylpropanoid biosynthesis, and thiamine metabolism were also significantly enriched. Among these, tryptophan metabolism showed the highest enrichment, with SC displaying markedly higher levels of tryptophol, tryptamine, n-acetyl-5-hydroxytryptamine, and indole-3-acetic acid than FJ. Tryptophan metabolism is associated with the production of auxin and secondary metabolites, playing a crucial role in regulating growth and responding to stress [48] in *D. rubrovolvata*. The high concentrations of 4-aminobenzoic acid, L-malic acid, and 4-hydroxybenzaldehyde in SC are associated with the crucial role of phenylpropanoid biosynthesis in producing aromatic compounds and metabolites involved in defense [49]. Thiamine metabolism was recognized as a crucial pathway that differentiates the origins, with pyruvic acid being reduced in SC and 4-methyl-5-thiazoleethanol being increased. The metabolic fluxes of amino acids, secondary metabolites, and vitamin metabolism differed between SC and FJ. Distinct metabolic signatures were observed between the SC and GZ groups (Figure 6F). Glycerophospholipid metabolism, two-component systems, and tryptophan metabolism were identified as crucial pathways, with glycerophospholipid metabolism being the most significant due to its notable accumulation in the SC. LysoPCs and phosphocholine were significantly upregulated in SC compared to GZ, suggesting active lipid remodeling [50]. The two-component system pathway showed high levels of glutamine, glutamic acid, and glucose 6-phosphate in SC. Tryptophol, tryptamine, and N-acetyl-5-hydroxytryptamine were upregulated in SC in the context of tryptophan metabolism, whereas metabolites associated with the kynurenine pathway were downregulated.

We identified variations in metabolic processes between GZ and FJ (Figure 6G), particularly in alkaloid biosynthesis and secondary metabolism with the main pathway of alkaloids from nicotinic acid, ornithine, and lysine. Within these pathways, intermediates of the TCA cycle (e.g., cis-aconitic acid, L-malic acid, and pipecolic acid) were upregulated in GZ. Significant differences were observed in glycerophospholipid metabolism, with the downregulation of key lipids (e.g., phosphocholine, 1-hexadecanoyl-sn-glycero-3-phosphocholine, and 1-stearoyl-sn-glycero-3-phosphocholine). The kynurenic acid content in GZ increased, accompanied by a rise in indole-3-acetic acid and tryptophol within the tryptophan metabolism pathway, indicating that tryptophan is metabolized differently based on geographical location.

These pathway-level differences collectively suggest that geographical origin is associated with coordinated metabolic reprogramming rather than isolated alterations in individual compounds. Notably, tryptophan metabolism was consistently enriched across multiple pairwise comparisons, indicating its potential role as a central metabolic axis underlying origin-dependent variation in *D. rubrovolvata*. Concurrent shifts in tryptophol, tryptamine, N-acetyl-5-hydroxytryptamine, indole-3-acetic acid, and kynurenine-related metabolites suggest that different geographical origins may influence the partitioning of tryptophan-derived metabolic flux into distinct downstream branches. Furthermore, the enrichment of phenylpropanoid biosynthesis, glycerophospholipid metabolism, and thiamine metabolism implies that differences among origins also involve aromatic compound biosynthesis, membrane lipid remodeling, and cofactor-related metabolism. These findings collectively support the notion that geographical origin may shape the metabolite profile of *D. rubrovolvata* through integrated effects on primary metabolism, secondary metabolism, and cellular adaptation processes.

## 4. Conclusions

In this study, we conducted a comprehensive analysis of the metabolite profiles of *D. rubrovolvata* collected from three different geographical locations using a UHPLC-Q-TOF-MS/MS-based metabolomics approach. A total of 383 metabolites were identified, with lipids and organic acids being the main components. Multivariate statistical analysis revealed notable differences in metabolomic profiles across various geographical samples. With a criterion of VIP > 1, FC > 2 or <0.5, and *p* < 0.05, 17 metabolites were identified as key differential metabolites to distinguish the three origins. The findings elucidated the interrelationships among these metabolites, identifying tryptophan and glycerophospholipid metabolism as the most significant pathways involved. Geographical origin is a critical determinant of the nutritional and chemical quality of *D. rubrovolvata*. Our research provides insights into *D. rubrovolvata* quality and geographic variation, offering a valuable reference for assessing the source traceability and quality. And it is imperative to conduct further research with a larger and more diverse sample set from various geographic regions to validate and quantitatively characterize the potential biomarkers associated with different production areas.

## Data Availability

The original contributions presented in this study are included in the article/Supplementary Materials. Further inquiries can be directed to the corresponding authors.

## Supplementary Materials

The following supporting information can be downloaded at: https://www.mdpi.com/article/10.3390/foods15081372/s1, Figure S1: Quality control assessment of data correction and analytical stability. Pearson correlation analysis of QC samples (A). The relative standard deviation of QC samples (B); Figure S2: Dynamic distribution diagrams of metabolite content differences between SC and FJ (A), SC and GZ (B), and GZ and FJ (C); Table S1: The statistical parameters of OPLS-DA modes of samples from three geographical regions of China; Table S2: Differential metabolites in the group of SC vs. FJ; Table S3: Differential metabolites in the group of SC vs. GZ; Table S4: Differential metabolites in the group of GZ vs. FJ; Table S5: Key differential metabolites in *D. rubrovolvata* samples from three geographical regions of China.

## Abbreviations

The following abbreviations are used in this manuscript:

ACN	acetonitrile
FJ	Fujian
GZ	Guizhou
KEGG	Kyoto Encyclopedia of Genes and Genomes
MeOH	methanol
OPLS-DA	orthogonal partial least-squares discriminant analysis
PCA	Pareto-scaled principal component analysis
QC	quality control
SC	Sichuan
UHPLC-Q-TOF-MS/MS	Ultra-high-performance liquid chromatography-quadrupole time-of-flight mass spectrometry
VIP	variable importance in the projection
