# Supplementary material for: Metabolomics Analysis Based on UHPLC-Q-TOF-MS/MS to Discriminate Dictyophora rubrovolvata from Different Geographical Origins of China
Source: Foods. 2026 Apr 15;15(8):1372. doi: 10.3390/foods15081372 (PMC13114384; doi:10.3390/foods15081372)
Supplement: Supplementary file 1 [file foods-15-01372-s001.zip › foods-4198120-supplementary figures.pdf]

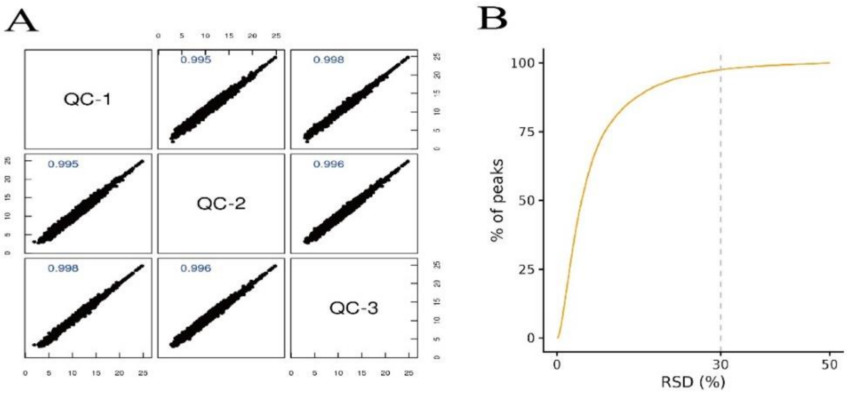

Figure S1. Quality control assessment of data correction and analytical stability.
Pearson correlation analysis of QC samples (A). The relative standard deviation of QC samples (B).

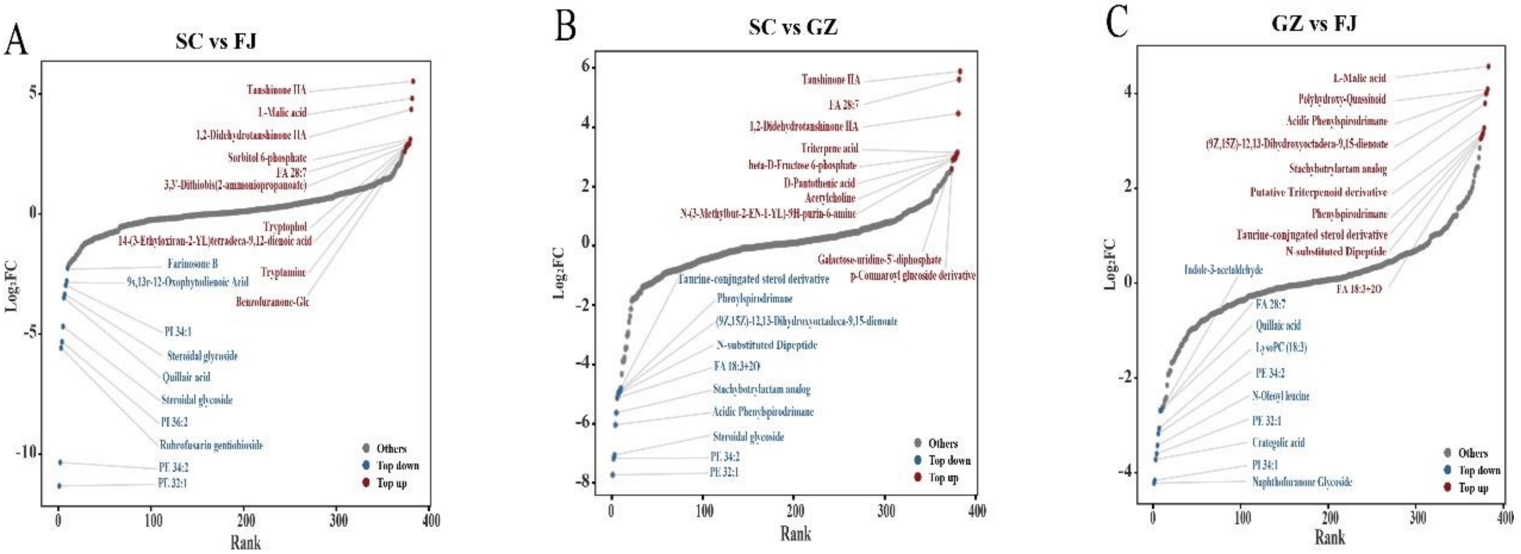

Figure S2. Dynamic distribution diagrams of metabolite content differences
between SC and FJ (A), SC and GZ (B), and GZ and FJ (C).
